# PDX1-engineered embryonic stem cell-derived insulin producing cells regulate hyperglycemia in diabetic mice

**DOI:** 10.1186/2047-1440-1-19

**Published:** 2012-10-18

**Authors:** Sudhanshu P Raikwar, Nicholas Zavazava

**Affiliations:** 1 Department of Internal Medicine, Division of Immunology, Roy J. and Lucille A. Carver College of Medicine, University of Iowa and Iowa City Veterans Affairs Medical Center, Building 41, Room #128, 601 Highway 6W, Iowa City, IA 52246, USA

**Keywords:** Bioluminescence imaging, Embryonic stem cells, Diabetes, Differentiation, Hyperglycemia, Insulin producing cells, Luciferase, Pancreatic and duodenal homeobox gene 1, Transplantation, Teratoma

## Abstract

**Background:**

Type 1 diabetes can be treated by the transplantation of cadaveric whole pancreata or isolated pancreatic islets. However, this form of treatment is hampered by the chronic shortage of cadaveric donors. Embryonic stem (ES) cell-derived insulin producing cells (IPCs) offer a potentially novel source of unlimited cells for transplantation to treat type 1 and possibly type 2 diabetes. However, thus far, the lack of a reliable protocol for efficient differentiation of ES cells into IPCs has hindered the clinical exploitation of these cells.

**Methods:**

To efficiently generate IPCs using ES cells, we have developed a double transgenic ES cell line R1Pdx1AcGFP/RIP-Luc that constitutively expresses pancreatic β-cell-specific transcription factor pancreatic and duodenal homeobox gene 1 (Pdx1) as well as rat insulin promoter (RIP) driven luciferase reporter. We have established several protocols for the reproducible differentiation of ES cells into IPCs. The differentiation of ES cells into IPCs was monitored by immunostaining as well as real-time quantitative RT-PCR for pancreatic β-cell-specific markers. Pancreatic β-cell specific RIP became transcriptionally active following the differentiation of ES cells into IPCs and induced the expression of the luciferase reporter. Glucose stimulated insulin secretion by the ES cell-derived IPCs was measured by ELISA. Further, we have investigated the therapeutic efficacy of ES cell-derived IPCs to correct hyperglycemia in syngeneic streptozotocin (STZ)-treated diabetic mice. The long term fate of the transplanted IPCs co-expressing luciferase in syngeneic STZ-induced diabetic mice was monitored by real time noninvasive *in vivo* bioluminescence imaging (BLI).

**Results:**

We have recently demonstrated that spontaneous *in vivo* differentiation of R1Pdx1AcGFP/RIP-Luc ES cell-derived pancreatic endoderm-like cells (PELCs) into IPCs corrects hyperglycemia in diabetic mice. Here, we investigated whether R1Pdx1AcGFP/RIP-Luc ES cells can be efficiently differentiated *in vitro* into IPCs. Our new data suggest that R1Pdx1AcGFP/RIP-Luc ES cells efficiently differentiate into glucose responsive IPCs. The ES cell differentiation led to pancreatic lineage commitment and expression of pancreatic β cell-specific genes, including Pax4, Pax6, Ngn3, Isl1, insulin 1, insulin 2 and PC2/3. Transplantation of the IPCs under the kidney capsule led to sustained long-term correction of hyperglycemia in diabetic mice. Although these newly generated IPCs effectively rescued hyperglycemic mice, an unexpected result was teratoma formation in 1 out of 12 mice. We attribute the development of the teratoma to the presence of either non-differentiated or partially differentiated stem cells.

**Conclusions:**

Our data show the potential of Pdx1-engineered ES cells to enhance pancreatic lineage commitment and to robustly drive the differentiation of ES cells into glucose responsive IPCs. However, there is an unmet need for eliminating the partially differentiated stem cells.

## Background

Type 1 diabetes is an autoimmune disease that can be surgically corrected by the transplantation of either the whole pancreas or that of pancreatic islets [1-4]. However, chronic shortage of organ donors, lifelong immunosuppressive therapy and chronic graft rejection currently limit the therapeutic potential of both options. Ultimately, chronic graft rejection leads to insulin dependence and the development of serious diabetic complications [5-10]. With the incidence of diabetes increasing at an alarming rate worldwide, there is an urgent and compelling need to develop novel treatment approaches. In this regard, embryonic stem (ES) cells and the recently developed induced pluripotent stem (iPS) cells potentially offer a novel alternative for the development of stem cell-based therapies [11-13]. However, the generation of functional insulin producing cells (IPCs) from ES or iPS cells has been rather inefficient; thus there is a need to improve on the protocols for the differentiation process.

Published work by others appeared to suggest that it is feasible to directly differentiate ES cells into IPCs [14]. Unfortunately, this work has remained irreproducible in many laboratories around the world. During embryonic development, a wide variety of transcription factors, including Pdx1, Ngn3, NeuroD, MafA, MafB, Gata4, Gata6, Ptf1a and Pax4 are involved in pancreatic β cell development and function. Pdx1 is the master regulator of pancreatic development [15-18] during embryogenesis, β cell differentiation [16,19-21] and is essential for the maintenance of β-cell function in the adults [15,16,22,23]. Mutations in the Pdx1 coding sequence in humans and mice lead to failure of the pancreatic organ to develop [25]. Here, we hypothesized that pancreatic lineage commitment of ES cells by pancreatic β cell-specific transcription factor Pdx1 enhances the generation of IPCs, thereby providing an unlimited source of cells for the treatment of diabetes. The rationale underlying this approach is that expression of pancreatic β cell-specific transcription factor Pdx1 will maximize the pancreatic lineage commitment and differentiation of ES and iPS cells into IPCs [15,17,26-31]. To facilitate greater efficiency of the generation of IPCs from ES cells, we have generated a double transgenic ES cell line R1Pdx1AcGFP/RIP-Luc to stably express an in-frame Pdx1AcGFP fusion protein [32]. Here, we describe the generation of IPCs *in vitro* using ES cells ectopically expressing Pdx1. For the real-time non-invasive *in vivo* bioluminescence imaging (BLI), we engineered a rat insulin promoter (RIP) driven luciferase reporter to monitor the fate and function of the IPCs post transplantation. Further, we show that transplantation of ES cell-derived IPCs efficiently corrects hyperglycemia in diabetic mice. However, the lack of cell surface markers specific for IPCs raises the potential for teratoma formation by residual non-differentiated ES cells. These studies justify the need to develop novel strategies for ES cell differentiation and purification of IPCs prior to transplantation.

## Materials and methods

### Cell lines

We have recently described the generation and characterization of the double transgenic mouse ES cell line R1Pdx1AcGFP/RIP-Luc stably expressing an in-frame Pdx1AcGFP fusion protein and RIP driven luciferase reporter in detail elsewhere [32]. The R1Pdx1AcGFP/RIP-Luc mouse ES cell line was maintained in DMEM containing 1,000 IU/ml leukemia inhibitory factor (LIF, ESGRO, ESG1107, Chemicon International Inc. Millipore, Billerica, MA, USA) and 15% fetal bovine serum (FBS), on primary murine embryonic fibroblast feeder layer as described earlier [33].

### *In vitro* differentiation of ES cells into IPCs

**Figure 1:**
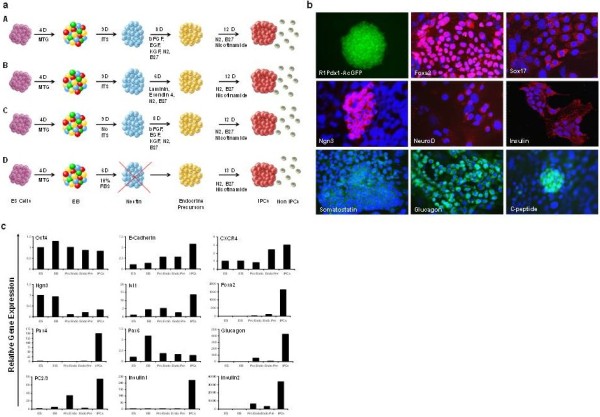
**Pdx1 promotes ES cell-differentiation into IPCs. (a) **Schematic representation of the ES cell differentiation protocols. **(b) **ES cells were subjected to differentiation using a multi-step differentiation protocol. The Pdx1 expressing R1Pdx1AcGFP/RIP-Luc ES cells (20X magnification) were subjected to EB formation followed by differentiation using four different protocols. Immunostaining of ES cell-derived IPCs for pancreatic markers: The Pdx1 expressing ES cell-derived differentiating cells and IPCs using protocol D were characterized by confocal microscope (60X magnification) following immunostaining using Foxa2, Sox17, Ngn3, NeuroD, insulin, somatostatin, glucagon and C-peptide. The results indicate that the differentiating cells express Foxa2, Sox17, Ngn3, and NeuroD which are critical for the development of IPCs. The differentiation leads to the generation of IPCs and glucagon expressing isolated cell clusters. As compared to the IPCs there is an abundance of glucagon expressing cells. **(c) **The *in vitro *differentiation of R1Pdx1AcGFP/RIP Luc ES cells into IPCs was monitored by real-time quantitative RT-PCR at various stages of differentiation. As a positive control, 18S rRNA was used to normalize and validate the results. The data indicate progressive up-regulation of Foxa2, Isl1, Pax4, glucagon, insulin 1, insulin 2 and PC2/3 genes following the differentiation of ES cells into IPCs.

We tested the *in vitro* differentiation of the R1Pdx1AcGFP/RIP-Luc ES cell line to generate glucose responsive IPCs using four modified protocols as depicted in Figure 1a as follows: (A) Undifferentiated R1Pdx1AcGFP/RIP-Luc ES cells were subjected to differentiation using a multi-step protocol [14]. Briefly, actively proliferating R1Pdx1AcGFP/RIP-Luc ES cells were trypsinized and 1 × 10^7^ cells were plated on to ultra-low attachment culture dishes in the presence of freshly prepared (45 μl/50 ml) 1:10 α-Monothioglycerol (Sigma Chemical Company, St. Louis, MO, USA) to promote embryoid body (EB) formation for four days (Figure 1a). The EBs were trypsinized and grown in serum-free DMEM supplemented with ITS-G (Invitrogen, Carlsbad, CA, USA) and enriched for nestin^+^ cells for nine days. The nestin^+^ cells were grown in DMEM/F12 (1:1) medium supplemented with 25 ng/ml bFGF (R&D System, Inc., Minneapolis, MN, USA), N2, B27, 10 ng/ml EGF and KGF supplements and cultured for eight days. The endocrine precursors obtained at the end of this stage were further propagated in low glucose DMEM supplemented with N2, B27 and 10 mM Nicotinamide to enrich IPCs for 12 days. (B) Day 4 EBs were cultivated in serum free DMEM with ITS-G (Invitrogen) for nine days followed by differentiation for six days in the presence of N2, B27, laminin and Exendin 4 and then similar to protocol A. (C) Day 4 EBs were cultivated in serum free DMEM similar to protocol A for nine days but without ITS-G. Subsequently the cells were cultured for 12 days as in protocol A. We also developed a new protocol (D) which completely eliminates the enrichment of the nestin^+^ cells. In the new protocol, the Day 4 EBs were directly cultivated in DMEM supplemented with 10% FBS for six days. The resulting cell population was subjected to differentiation in low glucose DMEM in the presence of N2, B27 and 10 mM nicotinamide for 13 days.

### Analysis of gene expression

To determine gene expression profiles at various stages of differentiation, total RNA was isolated from undifferentiated ES cells, EBs, proendocrine precursors, endocrine precursors and IPCs using the Absolute RNA Miniprep Kit (Stratagene, La Jolla, CA, USA) as per the instructions provided by the manufacturer. The cDNA synthesis was performed by reverse transcription using Stratascript QPCR cDNA synthesis kit (Stratagene) using the following conditions: Primer annealing at 25°C for 5 minutes, cDNA synthesis at 42°C for 45 minutes followed by reaction termination at 95°C. Real-time quantitative RT-PCR for various genes and the control 18S rRNA was performed with Brilliant SYBR Green QPCR master mix (Stratagene) with the following conditions: 1 denaturation cycle for 10 min at 95°C followed by 30-40 cycles (15-25 cycles for 18S rRNA) of 30 seconds denaturation, 1 minute annealing at 55-60°C and 1 minute extension at 72°C. The number of cycles, annealing temperature and extension time were optimized for the abundance of the transcripts, the *T*_*m*_ of the primers and the size of the amplicons. All reactions were performed in a Mx3000p Multiplex Quantitative PCR system (Stratagene). The dissociation curves were generated for all samples and the relative gene expression was normalized with the control 18S rRNA. The threshold cycle C_t_ value was determined as described [34]. Real-time quantitative RT-PCR reactions were performed multiple times to validate our results using the PCR primers listed in Table 1. The representative results of the real-time quantitative RT-PCR following ES cell differentiation using protocol D are presented in Figure 1c since the IPCs generated using the protocol D consistently expressed the highest levels of insulin.

**Table 1:** Nucleotide sequence of the PCR primers

**Gene**	**Forward Primer**	**Reverse Primer**
Oct4	5′GGCGTTCTCTTTGGAAAGGTGTTC3′	5′CTCGAACCACATCCTTCTCT3′
E-cadherin	5′AAACTTGGGGACAGCAACATCAG3′	5′TCTTTTGGTTTGCAGAGACAGGG3′
CXCR4	5′CGGGATGAAAACGTCCATTT3′	5′ATGACCAGGATCACCAATCCA3′
Ngn3	5′TGGCACTCAGCAAACAGCGA3′	5′ACCCAGAGCCAGACAGGTCT3′
Isl1	5′AGATATGGGAGACATGGGCGAT3′	5′ACACAGCGGAAACACTCGATG3′
Foxa2	5′ACCTGAGTCCGAGTCTGACC3′	5′GGCACCTTGAGAAAGCAGTC3′
Pax4	5′AAATGGCGCAGGCAAGAGAA3′	5′ATGAGGAGGCCACAGGA3′
Pax6	5′CAGTCACAGCGGAGTGAATC3′	5′CGCTTCAGCTGAAGTCGCAT3′
Glucagon	5′ACTCACAGGGCACATTCACC3′	5′CCAGTTGATGAAGTCCCTGG3′
Insulin 1	5′CCAGCTATAATCAGAGACCA3′	5′GTGTAGAAGAAGCCACGCT3′
Insulin 2	5′TCCGCTACAATCAAAAACCAT3′	5′GCTGGGTAGTGGTGGGTCTA3′
PC2/3	5′AGAGATTCCATTGTGTGGGA3′	5′CAAAATGGACTTGGTGCCCA3′
18S rRNA	5′TAACGAACGAGACTCTGGCAT3′	5′CGGACATCTAAGGGCATCACAG3′

### Immunostaining and immunohistochemistry

The ES cells grown on chambered glass slides were differentiated using the highly efficient protocol D and were fixed at different stages of differentiation with 2% paraformaldehyde, quenched in PBS containing 30 mM glycine and permeabilized with 0.1% Triton X-100 for 30 minutes at RT and blocked for 2 hours in PBS containing 2% Bovine Serum Albumin fraction V. The cells were stained with primary antibodies (1:250 dilution) against Ngn3 (SC-13794, goat polyclonal IgG, Santa Cruz Biotechnology, Santa Cruz, CA, USA), Foxa2 (SC-6554, goat polyclonal IgG, Santa Cruz Biotechnology), Sox17 (SC-17356, goat polyclonal IgG, Santa Cruz Biotechnology), glucagon (SC-13091, rabbit polyclonal IgG, Santa Cruz Biotechnology), somatostatin (SC-20999, rabbit polyclonal IgG, Santa Cruz Biotechnology) insulin (SC-7838, goat polyclonal IgG, Santa Cruz Biotechnology) and C-peptide (4593, rabbit polyclonal IgG, Cell Signaling Technology, Inc., Danvers, MA, USA), respectively. The cells were visualized by the use of either the Alexa Fluor 488 conjugated donkey anti-rabbit (A21206, Molecular Probes, Invitrogen,) or Alexa Fluor 546 conjugated donkey anti-goat (A11056, Molecular Probes, Invitrogen) secondary antibodies (1:500 dilution) and Vectashield mounting medium with DAPI (H1200, Vector laboratories, Inc. Burlingame, CA, USA). Multiphoton imaging was performed either on Radience 2100MP multiphoton microscope (Bio-Rad Laboratories, Hercules, CA, USA) or Zeiss LSM 710 inverted Axio Observer microscope (Carl Zeiss Microimaging GmbH, Jena, Germany) using 60X oil immersion objective lens and the images were captured as grayscale pictures and processed using the Image J program (National Institutes of Health) or ZEN Lite 2011 software (Carl Zeiss Microimaging GmbH, Jena, Germany). Immunohistochemical analysis and hematoxylin-eosin staining of the tissue sections was performed as described elsewhere [32].

### Measurement of insulin by ELISA

To test the glucose stimulated insulin secretion by ES cell-derived IPCs we used Mercodia Ultrasensitive Mouse Insulin ELISA, a solid phase two-site enzyme immunoassay (Mercodia, Inc., Winston Salem, NC, USA) [27]. This ELISA is a direct sandwich ELISA in which two distinct monoclonal antibodies are directed against two separate antigenic determinants on the insulin molecule. To measure insulin secretion by the ES cell-derived IPCs using different protocols, the cell culture supernatants in triplicates from 1 × 10^6^ ES cell-derived IPCs plated in six-well plates were collected 24 hours after changing the medium and were directly assayed using the mouse ultrasensitive insulin ELISA kit (Mercodia, Inc) and the values obtained were represented as insulin secretion (ng/mg) after normalizing to the total protein content (Protein assay reagent, Bio-Rad Laboratories).

For glucose stimulated insulin secretion studies, the cell culture supernatants in triplicates were collected from 5 × 10^4^ ES cell-derived IPCs that were plated in 24-well plates and incubated in KRBH buffer pH 7.4 (NaCl 118.4 mM, KCl 4.7 mM, KH_2_PO_4_ 1.2 mM, CaCl_2_ 2.4 mM, MgSO_4_ 1.2 mM, NaHCO_3_ 20 mM and HEPES 10 mM) in the presence of low glucose (2.8 mM) or high glucose (20 mM) and either with agonist tolbutamide (100 μM) or the antagonist nifedipine (50 μM) for 60 minutes. During incubation, the insulin present in the cell culture supernatant reacts with the peroxidase-conjugated anti-insulin antibodies and anti-insulin antibodies bound to the microplate well. Subsequent washing removes unbound enzyme labeled antibody. The bound conjugate is detected by a reaction with 3,3′5,5′-tetramethylbenzidine. The reaction is finally terminated by adding acid to give a colorimetric endpoint which is analyzed by an ELISA plate reader. The values obtained were represented as insulin secretion (ng/ml/hour).

### Mice

All animal experiments were approved and performed according to International Animal Care and Use Committee (IACUC) guidelines. The University of Iowa animal vivarium is accredited by the Association for the Assessment and Accreditation of Laboratory Animal Care (AAALAC). Eight week-old 129/SvJ mice (Jackson Laboratory, Bar Harbor, ME, USA) were used for the syngeneic transplantation experiments. Diabetes was induced by five consecutive intraperitoneal streptozotocin (STZ) (EMD Millipore Corporation, Billerica, MA< USA) injections (50 mg/Kg body weight). STZ was reconstituted in ice cold fresh sodium citrate buffer (pH 4.5) immediately prior to injection. The fasting blood glucose levels were regularly monitored using a HemoCue glucose 201 analyzer (HemoCue AB, Ängelholm, Sweden). Mice with blood glucose levels >350 mg/dl for two consecutive readings that were five days apart were considered diabetic. Diabetic 129/SvJ mice do not survive beyond 15 days due to severe hyperglycemia. Approximately 1 × 10^6^ R1Pdx1AcGFP ES cell-derived IPCs were transplanted under the kidney capsule of the diabetic mice.

### Real-time noninvasive *in vivo* bioluminescence imaging (BLI)

The *in vivo* fate and function of the transplanted IPCs was monitored using real-time noninvasive *in vivo* BLI. The Xenogen IVIS Imaging System 200 Series (Caliper Life Sciences, Hopkinston, MA, USA) was used in these experiments as described elsewhere [32].

### Statistical analysis

The experimental data were analyzed using the GraphPad Prism 5 software (GraphPad Software, Inc., San Diego, CA, USA). The data were tested for significance with Student’s *t-*test or one-way ANOVA where applicable. In all cases, *P <0.05 was considered significant.

## Results

### ES cell-derived IPCs secrete insulin

The results from multiple earlier studies have indicated low insulin secretion by ES cell-derived IPCs [27,35-39]. Since Pdx1 is a major transcription factor for the development of the pancreas as well as pancreatic β cells, we reasoned that generating a stable Pdx1-expressing ES cell line R1Pdx1AcGFP/RIP-Luc and directing its differentiation towards IPCs could increase the yield of IPCs. To investigate which approach was more effective at generating IPCs, four different differentiation protocols as described in the methods section and depicted in Figure 1a were studied. The progressive differentiation of the R1Pdx1AcGFP/RIP-Luc ES cell line into IPCs was monitored by immunostaining for various pancreatic markers (Figure 1b). As expected the differentiation process led to the expression of insulin producing cell clusters. Our immunostaining results using the differentiation protocol D indicate that the cells express Foxa2, Sox17, Ngn3 and NeuroD markers during the early steps of differentiation (Figure 1b). In addition to C-peptide and insulin expressing cells, we found that a significant cell population expressed glucagon. The newly generated IPCs were organized into small islet-like clusters (Figure 1b). Based on immunostaining results, we estimated that approximately 25 to 30% of the cells were expressing insulin. To further confirm that the cells had differentiated into pancreatic lineage, the expression of various pancreatic genes was studied. We used real-time quantitative RT-PCR analysis on RNA isolated from the cells at the completion of various stages to monitor the *in vitro* differentiation of ES cells into IPCs (Figure 1c). The RT-PCR results from a representative differentiation using protocol D indicate a progressive up-regulation of pancreatic β-cell-specific genes commensurate with the differentiation status of the ES cells. A significant up-regulation of Foxa2, Pax4, insulin 1, insulin 2, PC2/3 and glucagon gene expression in the IPCs was observed.

**Figure 2:**
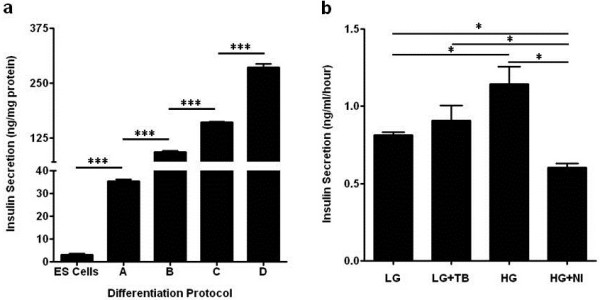
**Pdx1 expressing ES cell-derived IPCs are glucose responsive. (a) **Insulin secretion by 1 × 10^6^ IPCs plated in triplicates in six-well plates and generated using all the four differentiation protocols was evaluated by ELISA and expressed relative to the protein content of the cells. The results indicate that generation of IPCs using protocol D by eliminating the nestin selection stage significantly improves the generation of IPCs. The values represent mean ± SD (n = 3) where ***P <0.0001 vs other groups by one-way ANOVA with Tukey’s multiple pairwise comparison. **(b) **For testing glucose stimulated insulin secretion, 5 × 10^4^ IPCs were plated in 24-well plates in triplicates in the presence of 2.8 mM glucose (LG), 2.8 mM glucose + 100 μM tolbutamide (LG + TB), 20 mM glucose (HG) or 20 mM glucose + 50 μM nifedipine (HG + NI). The insulin secretion was monitored by an ultrasensitive ELISA. The values represent mean ± SE (n = 3) and all data were tested for significance with Student’s *t-*test or one way ANOVA where applicable. In all cases, *P <0.05 was considered statistically significant.

The insulin production by ES cell-derived IPCs was determined by an ultrasensitive ELISA. The ELISA results indicate that the ES cell-derived IPCs robustly secrete insulin (Figure 2a). The protocols A to C have slight variations but include the nestin selection stage while protocol D is devoid of the nestin selection stage. Our ELISA data suggest that elimination of the nestin selection stage (protocol D) significantly enhances the generation of IPCs as well as improves insulin secretion (Figure 2a). Further, we tested the glucose responsive insulin secretion by the IPCs in the presence of low and high glucose as well as low glucose plus agonist tolbutamide and high glucose plus antagonist nifedipine (Figure 2b). In the presence of low glucose, the insulin secretion is very low and marginally increases in the presence of the agonist tolbutamide. However, in the presence of high glucose the insulin secretion is comparatively higher and in the presence of high glucose and the antagonist nifedipine the insulin secretion is significantly inhibited as expected. These results confirm that the newly generated IPCs are indeed glucose responsive. All experiments discussed hereafter were performed with the IPCs generated following differentiation with protocol D.

### ES cell-derived IPCs significantly reduce hyperglycemia

**Figure 3:**
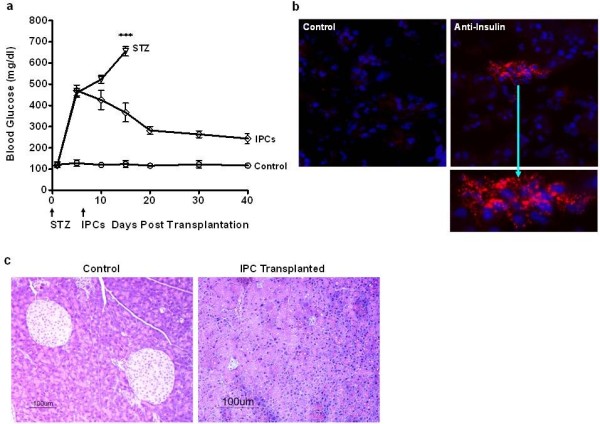
**R1Pdx1AcGFP ES cell-derived IPCs correct hyperglycemia in diabetic mice. (a) **The therapeutic efficacy of R1Pdx1AcGFP/RIP-Luc ES cell-derived IPCs was evaluated following transplantation under the kidney capsule of syngeneic 129/SvJ mice (n = 12) rendered diabetic by streptozotocin (STZ) treatment. The blood glucose levels continued to decline beyond Day 15 post-transplantation and stabilized thereafter. Normal nondiabetic mice (n = 5) and STZ-treated 129/SvJ mice (n = 5) were used as appropriate controls for comparing the blood glucose levels. As early as Day 15 and throughout the duration of the study, the blood glucose levels of mice transplanted with IPCs were significantly lower compared to the STZ-treated mice. The data were analyzed by one-way ANOVA and are presented as mean ± SD, ***P <0.0001, using Tukey’s pairwise multiple comparison for Day 15. **(b) **The kidney bearing the transplanted IPCs was evaluated for insulin expression by immunostaining using an anti-insulin antibody. The results indicate the localized presence of ES cell-derived insulin expressing cells. **(c) **Control mouse pancreas and the pancreas from IPCs transplanted mice 150 days post transplantation were examined by hematoxylin-eosin staining. Unlike control pancreas from non-diabetic mouse, the IPCs transplanted streptozotocin-treated diabetic mouse pancreas did not show any evidence of pancreatic beta cell regeneration.

To determine whether R1Pdx1AcGFP/RIP-Luc ES cell-derived IPCs correct hyperglycemia in diabetic mice, 1 × 10^6^ IPCs were transplanted under the kidney capsule in STZ-induced diabetic syngeneic 129/SvJ mice. Healthy mice have a fasting serum glucose level of about 105 ± 10 mg/dl. In contrast, our diabetic mice had elevated glucose levels in the range of 500 to 650 mg/dl. The IPC treatment group comprised of 12 mice while normal non-diabetic as well as STZ treated control group comprised of 5 mice each. Blood glucose levels were measured prior to IPCs transplantation and every three to five days post-IPCs transplantation. The STZ-treated diabetic control mice exhibited elevated blood glucose levels (>600 mg/dl), polyuria, weight loss, muscle wasting, hunched back posture, poor mobility, soiled body coat color, shivering and died within 14 days. The IPCs transplanted mice showed remarkable correction of the hyperglycemia as early as Day 15 post-transplantation. Further, these mice continued to exhibit near normoglycemic blood glucose levels beyond 40 days post-transplantation (Figure 3a). The Day 40 blood glucose values for the IPCs-treated mice reached the lower threshold of 250 mg/dl (mean 243.8 (95% CI 216.2 to 271.4)) remaining higher than that of control mice. Although complete correction of blood glucose levels was not observed, the IPC transplanted mice survived for >150 days post IPC transplantation. This finding suggests that our IPCs were not capable of fully reducing the blood glucose levels. With a significant improvement in the blood glucose levels, the IPC transplanted mice displayed improved feed consumption, improved posture, increase in body weights, reduced urination frequency, lack of shivering, improved mobility, shiny body coat color and survival throughout the duration of the study. To confirm that the IPCs transplanted under the kidney capsule were indeed responsible for the correction of hyperglycemia, the kidney sections from the nephrectomized mice were subjected to immunostaining using anti-insulin antibody. Our immunostaining results (Figure 3b) confirm the presence of insulin expressing cells in the transplanted kidney. Finally, to rule out the possibility of endogenous pancreatic β cell regeneration, we performed hematoxylin-eosin staining of the pancreas on Day 150 from the STZ treated mice that were transplanted with IPCs. Our histopathology data (Figure 3c) demonstrates that the normal pancreas has extremely large pancreatic islets. However, the pancreas from the mice transplanted with IPCs revealed near complete loss of pancreatic islets and no evidence of endogenous pancreatic β-cell regeneration. Taken together, our data suggest that ES cell-derived IPCs were indeed responsible for the long-term survival and correction of the diabetic phenotype post-transplantation in the diabetic mice.

### *In vivo* imaging of the IPCs using real-time noninvasive bioluminescence imaging (BLI)

**Figure 4:**
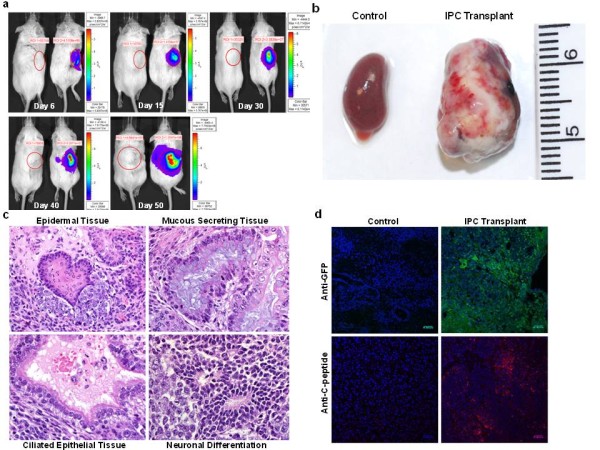
**Pdx1 expressing ES cell-derived IPCs cause teratoma formation. (a) **The long-term fate of the IPCs transplanted under the renal capsule in diabetic mice was monitored by real-time noninvasive bioluminescence imaging (BLI). Our ES cells were engineered using rat insulin promoter (RIP) driven luciferase reporter. Following differentiation of the ES cells, the transcriptional activation of the RIP leads to a selective robust expression of luciferase in the IPCs. In one of the IPC transplanted mouse, the BLI signal showed a progressive increase, especially beyond Day 40 post-transplantation with maximum BLI signal intensity at Day 50 post-transplantation. **(b) **The mouse transplanted with ES cell-derived IPCs was sacrificed and the internal organs were examined. The kidney bearing the transplanted IPCs revealed the presence of teratoma formation covering the dorsal and ventral sides of the kidney. **(c) **Histopathology following hematoxylin-eosin staining revealed the presence of epidermal, mucous secreting, ciliated epithelial as well as the neuronal tissues. However, there was no evidence of any metastatic lesions in any other organs. **(d)** Immunostaining using anti-GFP and anti-C-peptide antibodies revealed the presence of both AcGFP and insulin-positive transplanted cells.

One of the major caveats in the field of islet transplantation is the inability to successfully and noninvasively monitor the fate and function of the transplanted islets. We reasoned that incorporation of a tissue-specific promoter into the IPCs to drive the expression of luciferase reporter would enable us to noninvasively monitor their long-term survival and function *in vivo*. This was achieved by engineering RIP driven luciferase reporter in the undifferentiated R1Pdx1AcGFP-expressing ES cells [32]. In the undifferentiated state, the RIP is transcriptionally inactive as determined by the luciferase assay. However, in the R1Pdx1AcGFP/RIP-Luc ES cell-derived IPCs, the RIP becomes transcriptionally active and drives a high level expression of the luciferase gene. Real time *in vivo* BLI of the mice transplanted with R1Pdx1AcGFP ES cell-derived IPCs revealed a progressive increase in the luciferase activity with time (Figure 4a). However, in 1 of the 12 transplanted mice, BLI signal intensity continued to increase in size as well as magnitude beyond 30 days post-transplantation. We further followed this observation by repeating BLI on Day 50 post-transplantation. At this time point, the BLI signal intensity was saturated and dispersed beyond the transplanted area (Figure 4a). To investigate the underlying reason, we sacrificed this mouse at Day 52 and performed necropsy. We discovered that the transplanted IPCs had formed a large teratoma in the kidney (Figure 4b). This finding, although not entirely surprising, indicated that some of the ES cells remained in the non-differentiated state or were only partially differentiated and formed teratomas. Prior to transplantation, non-differentiated cells could not be removed due to a lack of β-cell-specific surface markers to selectively enrich the IPCs.

Histopathological examination of the teratoma revealed the presence of epidermoid, mucous secreting, ciliated epithelial as well as neuronal cells (Figure 4c). Finally, to confirm the presence of IPCs in the kidney bearing teratoma, immunostaining was performed. Insulin staining cells were detectable (Figure 4d) in the kidney containing IPCs but not in control kidneys from the contra-lateral side, suggesting that the transplanted cells were producing insulin under the renal capsule. Taken together, our results highlight the potential of ES cells to generate IPCs that can be used therapeutically. However, the differentiation process is not yet optimal since one of the IPC-transplanted mouse developed a teratoma.

## Discussion

ES and iPS cells could potentially overcome the chronic shortage of cadaveric organs if we are able to establish efficient differentiation protocols. Derivation of specific cell types and tissues from ES and iPS cells could end the need for donated cadaveric tissue. However, the current protocols for the *in vitro* differentiation of ES and iPS cells into IPCs are still very inefficient, time consuming and very expensive. Currently, there are two different approaches that allow the generation of IPCs using ES cells: (a) embryoid body (EB) formation [40] and (b) definitive endoderm (DE) formation [32]. Here, we investigated the generation of IPCs using the EB formation approach. The EB represents an early differentiation stage that is characterized by the formation of a three-dimensional cluster of cells, which give rise to tissues representing all the three primary germ layers.

A number of differentiation protocols for the generation of IPCs using ES cells have been reported [14,27,36,38,41-49]. However, a major caveat is the lack of pancreatic β-cell-specific surface markers that allow purification of the IPCs. A second obstacle is that the ES cell differentiation procedures used are extremely inefficient. Consequently, in some of these studies, ES cells could be differentiated into IPCs but their transplantation in diabetic mice seldom led to a correction of the hyperglycemic state [27,37,38,46]. Moreover, the insulin production by the ES cell-derived IPCs has been recently challenged and suggested to be rather an artifact because the cells could uptake insulin from the culture media and release it when they apoptose [50,51], leading to false positive results.

The embryonic development of pancreatic β cells requires regulated expression of multiple transcription factors. However, Pdx1 has been shown to be the master regulator of the pancreas development and plays a crucial role during the development and function of pancreatic β cells [16,17,25,28]. Recently, ectopic Pdx1 expression in bone marrow derived mesenchymal stromal cells, human ES cells and adipose tissue derived stem cells has been demonstrated to promote their differentiation into IPCs [26,52-55]. One of the critical steps in the differentiation of ES cells into IPCs is the derivation of the Pdx1 expressing pancreatic progenitors. We, therefore, hypothesized that ectopic over-expression of Pdx1 in ES cells *in vitro* enforces lineage commitment and progressive differentiation of ES cells into IPCs. Here, we report an alternative approach for the efficient differentiation of murine ES cells into IPCs. The unique features about our differentiation strategy are the use of the pancreatic β-cell-specific transcription factor Pdx1 and the development of a new protocol that eliminates the nestin selection stage, thus reducing the overall differentiation time.

Here, we employed a multistep differentiation strategy to study and optimize the differentiation of mouse ES cells into IPCs. The R1Pdx1AcGFP/RIP-Luc ES cell line was subjected to *in vitro* differentiation using the EB formation protocol. We compared four different protocols with modifications post-EB formation for the directed differentiation of the R1Pdx1AcGFP/RIP-Luc ES cells into IPCs. The gene expression analysis of the ES cells undergoing differentiation into IPCs indicates a selective up-regulation of pancreatic β-cell-specific genes, including Pax4, Isl1, insulin 1, insulin 2 and PC2/3, thereby implying a lineage commitment towards β-cells. These findings support the view that ES cells can be coaxed to differentiate into physiologically responsive IPCs. Our immunostaining results indicate that the ES cells undergoing differentiation express Foxa2, Sox17, Ngn3 and NeuroD, as well as C-peptide. These results confirm that our differentiation protocol triggers a temporally regulated signaling cascade mediated by multiple pancreatic β-cell-specific transcription factors, which ultimately leads to the robust generation of IPCs. In some cases, the IPCs are typically arranged in the form of small pancreatic islet-like clusters.

Interestingly, despite ectopic Pdx1 expression in our R1Pdx1AcGFP/RIP-Luc ES cells, we found a significant number of glucagon-expressing cells in addition to the IPCs. Similar results were observed in transplanted diabetic mice following spontaneous *in vivo* differentiation of R1Pdx1AcGFP/RIP-Luc ES cell-derived PELCs [32]. However, at present we do not fully understand the molecular mechanism underlying the generation of glucagon expressing cells in our differentiation studies. Our results are not entirely surprising because in an earlier study, inducible biphasic expression of Pdx1 led to the differentiation of mouse ES cells into both insulin and glucagon expressing cells [26]. It is possible that sustained Pdx1 expression in our ES cells leads to the development of a bihormonal progenitor cell population during differentiation, which in turn gives rise to both insulin- and glucagon-expressing cells. These findings suggest that ectopic Pdx1 expression alone may not be sufficient to allow for the complete maturation of the IPCs *in vitro*. We, however, anticipate that these cells eventually mature *in vivo* post-transplantation and become mono-hormonal. In a recent study, adenoviral-mediated coexpression of Pdx1 and MafA with either Ngn3 or NeuroD has been shown to improve the generation of IPCs [56]. However, the critical transplantation experiments to demonstrate the therapeutic efficacy of the ES cell-derived IPCs were not performed.

The insulin secretion, as well as glucose responsiveness, of the R1Pdx1AcGFP/RIP-Luc ES cell-derived IPCs was confirmed by an ultrasensitive ELISA. Our ELISA data suggest that constitutive Pdx1 expression in our R1Pdx1AcGFP/RIP-Luc ES cell-derived IPCs leads to a robust glucose responsive insulin secretion as compared to the biphasic Pdx1 expression or combined inducible Pdx1 and Ngn3 expression as reported earlier [26,57]. It is possible that constitutive Pdx1 expression maintains the insulin processing and secretion mechanism in ES cell-derived IPCs in a dynamic state, thereby facilitating glucose responsive insulin secretion. Moreover, in these earlier studies, the much needed transplantation experiments to evaluate the therapeutic efficacy of ES cell-derived IPCs to correct hyperglycemia in diabetic mice were not performed.

The therapeutic efficacy of the IPCs derived using our new differentiation protocol was tested in streptozotocin-treated 129/SvJ syngeneic diabetic mice by transplanting the cells under the kidney capsule. The 129/SvJ mice administered STZ for five consecutive days become progressively diabetic, do not recover from severe hyperglycemia and die between Days 10 and 15 due to failure of endogenous β-cell regeneration as reported earlier [32]. In the present studies, R1Pdx1AcGFP/RIP-Luc ES cell-derived IPCs were transplanted under the kidney capsule of the syngeneic 129/SvJ diabetic mice. The blood glucose levels in mice transplanted with the IPCs demonstrated a steady decline until Day 20 when they finally stabilized throughout the duration of the study. However, complete normoglycemia was not observed in the IPCs transplanted mice. We speculate that the base-line hyperglycemia could easily be overcome by transplanting a greater number of IPCs. To further validate our results, we performed immunofluorescence analysis of the kidney that was transplanted with the IPCs. Our immunofluorescence data demonstrate the presence of positive insulin staining of the R1Pdx1AcGFP/RIP-Luc ES cell-derived IPCs in the kidney. Our results confirm that the transplanted IPCs were functional and able to correct hyperglycemia.

Thus, our studies highlight the need to develop novel strategies to selectively enrich IPCs by eliminating the tumor-causing cells prior to transplantation. Possible candidate cell surface molecules that could be used to remove non-differentiated and partially differentiated ES cells are CXCR4, CD326 and surface specific early antigen.

## Conclusions

Our current studies demonstrate the potential for pluripotent stem cell-based treatment of type 1 diabetes by enhanced generation of IPCs from ES cells. Although we achieved high level insulin secretion by our newly established protocol, we believe that ectopic Pdx1 expression in our ES cells alone may not be sufficient to robustly generate IPCs. Our studies also demonstrate the potential utility of real-time noninvasive BLI to monitor the *in vivo* fate of transplanted IPCs. Finally, our studies provide evidence that for more efficient application of ES cells in the treatment of diabetes we will need to be able to separate teratogenic non-differentiated ES cells from fully differentiated IPCs. While much remains to be done, ES and the iPS cells have the potential to become a new renewable source of IPCs that could alleviate the chronic shortage of pancreatic islets for the treatment of type 1 diabetes.

## Abbreviations

AAALAC: Association for the Assessment and Accreditation of Laboratory Animal Care; AcGFP: Aequorea coerulescens green fluorescent protein; BLI: bioluminescence imaging; DE: definitive endoderm; EB: embryoid body; ES cells: embryonic stem cells; IACUC: International Animal Care and Use Committee; IPCs: insulin producing cells; LIF: leukemia inhibitory factor; Luc: luciferase; Pdx1: pancreatic and duodenal homeobox gene 1; PELCs: pancreatic endoderm-like cells; RIP: rat insulin promoter; STZ: streptozotocin.

## Competing interest

SR and NZ do not have any commercial associations or non-financial competing interests and declare no conflicts of interest.

## Authors contributions

SPR contributed to the conception and design, collection and assembly of data, data analysis and interpretation, manuscript writing, and final approval of the manuscript. NZ contributed to the conception and design, data analysis and interpretation, manuscript editing, and final approval of the manuscript.

## References

[B1] Daneman D Type 1 diabetes Lancet 2006 367 847 858 10.1016/S0140-6736(06)68341-4 16530579

[B2] Eisenbarth GS Type I diabetes mellitus. A chronic autoimmune disease N Engl J Med 1986 314 1360 1368 10.1056/NEJM198605223142106 3517648

[B3] Shapiro AM Lakey JR Ryan EA Korbutt GS Toth E Warnock GL Kneteman NM Rajotte RV Islet transplantation in seven patients with type 1 diabetes mellitus using a glucocorticoid-free immunosuppressive regimen N Engl J Med 2000 343 230 238 10.1056/NEJM200007273430401 10911004

[B4] Shapiro AM Ricordi C Hering BJ Auchincloss H Lindblad R Robertson RP Secchi A Brendel MD Berney T Brennan DC Cagliero E Alejandro R Ryan EA DiMercurio B Morel P Polonsky KS Reems JA Bretzel RG Bertuzzi F Froud T Kandaswamy R Sutherland DE Eisenbarth G Segal M Preiksaitis J Korbutt GS Barton FB Viviano L Seyfert-Margolis V Bluestone J International trial of the Edmonton protocol for islet transplantation N Engl J Med 2006 355 1318 1330 10.1056/NEJMoa061267 17005949

[B5] Nathan DM Long-term complications of diabetes mellitus N Engl J Med 1993 328 1676 1685 10.1056/NEJM199306103282306 8487827

[B6] Nathan DM Finding new treatments for diabetes–how many, how fast… how good? N Engl J Med 2007 356 437 440 10.1056/NEJMp068294 17267901

[B7] Ricordi C Strom TB Clinical islet transplantation: advances and immunological challenges Nat Rev Immunol 2004 4 259 268 10.1038/nri1332 15057784

[B8] Ryan EA Lakey JR Rajotte RV Korbutt GS Kin T Imes S Rabinovitch A Elliott JF Bigam D Kneteman NM Warnock GL Larsen I Shapiro AM Clinical outcomes and insulin secretion after islet transplantation with the Edmonton protocol Diabetes 2001 50 710 719 10.2337/diabetes.50.4.710 11289033

[B9] Ryan EA Paty BW Senior PA Bigam D Alfadhli E Kneteman NM Lakey JR Shapiro AM Five-year follow-up after clinical islet transplantation Diabetes 2005 54 2060 2069 10.2337/diabetes.54.7.2060 15983207

[B10] Smith RN Kent SC Nagle J Selig M Iafrate AJ Najafian N Hafler DA Auchincloss H Orban T Cagliero E Pathology of an islet transplant 2 years after transplantation: evidence for a nonimmunological loss Transplantation 2008 86 54 62 18622278 10.1097/TP.0b013e318173a5da

[B11] Evans MJ Kaufman MH Establishment in culture of pluripotential cells from mouse embryos Nature 1981 292 154 156 10.1038/292154a0 7242681

[B12] Takahashi K Yamanaka S Induction of pluripotent stem cells from mouse embryonic and adult fibroblast cultures by defined factors Cell 2006 126 663 676 10.1016/j.cell.2006.07.024 16904174

[B13] Thomson JA Itskovitz-Eldor J Shapiro SS Waknitz MA Swiergiel JJ Marshall VS Jones JM Embryonic stem cell lines derived from human blastocysts Science 1998 282 1145 1147 9804556 10.1126/science.282.5391.1145

[B14] Lumelsky N Blondel O Laeng P Velasco I Ravin R McKay R Differentiation of embryonic stem cells to insulin-secreting structures similar to pancreatic islets Science 2001 292 1389 1394 10.1126/science.1058866 11326082

[B15] Ahlgren U Jonsson J Jonsson L Simu K Edlund H Beta-cell-specific inactivation of the mouse Ipf1/Pdx1 gene results in loss of the beta-cell phenotype and maturity onset diabetes Genes Dev 1998 12 1763 1768 10.1101/gad.12.12.1763 9637677 PMC316911

[B16] Holland AM Gonez LJ Naselli G MacDonald RJ Harrison LC Conditional expression demonstrates the role of the homeodomain transcription factor Pdx1 in maintenance and regeneration of beta-cells in the adult pancreas Diabetes 2005 54 2586 2595 10.2337/diabetes.54.9.2586 16123346

[B17] Offield MF Jetton TL Labosky PA Ray M Stein RW Magnuson MA Hogan BL Wright CV PDX-1 is required for pancreatic outgrowth and differentiation of the rostral duodenum Development 1996 122 983 995 8631275 10.1242/dev.122.3.983

[B18] Stoffers DA Heller RS Miller CP Habener JF Developmental expression of the homeodomain protein IDX-1 in mice transgenic for an IDX-1 promoter/lacZ transcriptional reporter Endocrinology 1999 140 5374 5381 10.1210/en.140.11.5374 10537169

[B19] Ferber S Halkin A Cohen H Ber I Einav Y Goldberg I Barshack I Seijffers R Kopolovic J Kaiser N Karasik A Pancreatic and duodenal homeobox gene 1 induces expression of insulin genes in liver and ameliorates streptozotocin-induced hyperglycemia Nat Med 2000 6 568 572 10.1038/75050 10802714

[B20] Heller RS Stoffers DA Bock T Svenstrup K Jensen J Horn T Miller CP Habener JF Madsen OD Serup P Improved glucose tolerance and acinar dysmorphogenesis by targeted expression of transcription factor PDX-1 to the exocrine pancreas Diabetes 2001 50 1553 1561 10.2337/diabetes.50.7.1553 11423476

[B21] Wang H Maechler P Ritz-Laser B Hagenfeldt KA Ishihara H Philippe J Wollheim CB Pdx1 level defines pancreatic gene expression pattern and cell lineage differentiation J Biol Chem 2001 276 25279 25286 10.1074/jbc.M101233200 11309388

[B22] Brissova M Shiota M Nicholson WE Gannon M Knobel SM Piston DW Wright CV Powers AC Reduction in pancreatic transcription factor PDX-1 impairs glucose-stimulated insulin secretion J Biol Chem 2002 277 11225 11232 10.1074/jbc.M111272200 11781323

[B23] Kulkarni RN Jhala US Winnay JN Krajewski S Montminy M Kahn CR PDX-1 haploinsufficiency limits the compensatory islet hyperplasia that occurs in response to insulin resistance J Clin Invest 2004 114 828 836 15372107 10.1172/JCI21845 PMC516265

[B24] Stoffers DA Zinkin NT Stanojevic V Clarke WL Habener JF Pancreatic agenisis attributable to a single nucleotide deletion in the human IPF1 gene coding sequence Nat Genet 1997 15 106 110 10.1038/ng0197-106 8988180

[B25] Jonsson J Carlsson L Edlund T Edlund H Insulin-promoter-factor 1 is required for pancreas development in mice Nature 1994 371 606 609 10.1038/371606a0 7935793

[B26] Bernardo AS Cho H Mason S Docherty HM Pedersen RA Vallier L Docherty K Biphasic induction of Pdx1 in mouse and human embryonic stem cells can mimic development of pancreatic beta cells Stem Cells 2008 27 341 351 19056911 10.1634/stemcells.2008-0310

[B27] Blyszczuk P Czyz J Kania G Wagner M Roll U St-Onge L Wobus AM Expression of Pax4 in embryonic stem cells promotes differentiation of nestin-positive progenitor and insulin-producing cells Proc Natl Acad Sci U S A 2003 100 998 1003 10.1073/pnas.0237371100 12525695 PMC298715

[B28] Oliver-Krasinski JM Kasner MT Yang J Crutchlow MF Rustgi AK Kaestner KH Stoffers DA The diabetes gene Pdx1 regulates the transcriptional network of pancreatic endocrine progenitor cells in mice J Clin Invest 2009 119 1888 1898 10.1172/JCI37028 19487809 PMC2701861

[B29] Raikwar SP Zavazava N Insulin producing cells derived from embryonic stem cells: Are we there yet? J Cell Physiol 2009 218 256 263 10.1002/jcp.21615 18932230 PMC2649936

[B30] Sosa-Pineda B Chowdhury K Torres M Oliver G Gruss P The Pax4 gene is essential for differentiation of insulin-producing beta cells in the mammalian pancreas Nature 1997 386 399 402 10.1038/386399a0 9121556

[B31] Alipio Z Liao W Roemer EJ Waner M Fink LM Ward DC Ma Y Reversal of hyperglycemia in diabetic mouse models using induced-pluripotent stem (iPS)-derived pancreatic beta-like cells Proc Natl Acad Sci U S A 2010 107 13426 13431 10.1073/pnas.1007884107 20616080 PMC2922145

[B32] Raikwar SP Zavazava N Spontaneous in vivo differentiation of embryonic stem cell-derived pancreatic endoderm-like cells corrects hyperglycemia in diabetic mice Transplantation 2011 91 11 20 10.1097/TP.0b013e3181fdd98b 21452407

[B33] Bonde S Zavazava N Immunogenicity and engraftment of mouse embryonic stem cells in allogeneic recipients Stem Cells 2006 24 2192 2201 10.1634/stemcells.2006-0022 16794265

[B34] Huang Y Kucia M Hussain LR Wen Y Xu H Yan J Ratajczak MZ Ildstad ST Bone marrow transplantation temporarily improves pancreatic function in streptozotocin-induced diabetes: potential involvement of very small embryonic-like cells Transplantation 2010 89 677 685 10.1097/TP.0b013e3181c9dc7d 20110858 PMC2844483

[B35] Sipione S Eshpeter A Lyon JG Korbutt GS Bleackley RC Insulin expressing cells from differentiated embryonic stem cells are not beta cells Diabetologia 2004 47 499 508 10.1007/s00125-004-1349-z 14968299

[B36] Soria B Roche E Berna G Leon-Quinto T Reig JA Martin F Insulin-secreting cells derived from embryonic stem cells normalize glycemia in streptozotocin-induced diabetic mice Diabetes 2000 49 157 162 10.2337/diabetes.49.2.157 10868930

[B37] Fujikawa T Oh SH Pi L Hatch HM Shupe T Petersen BE Teratoma formation leads to failure of treatment for type I diabetes using embryonic stem cell-derived insulin-producing cells Am J Pathol 2005 166 1781 1791 10.1016/S0002-9440(10)62488-1 15920163 PMC1602425

[B38] Hori Y Rulifson IC Tsai BC Heit JJ Cahoy JD Kim SK Growth inhibitors promote differentiation of insulin-producing tissue from embryonic stem cells Proc Natl Acad Sci U S A 2002 99 16105 16110 10.1073/pnas.252618999 12441403 PMC138572

[B39] Treff NR Vincent RK Budde ML Browning VL Magliocca JF Kapur V Odorico JS Differentiation of embryonic stem cells conditionally expressing neurogenin 3 Stem Cells 2006 24 2529 2537 10.1634/stemcells.2006-0082 16809427

[B40] Schroeder IS Rolletschek A Blyszczuk P Kania G Wobus AM Differentiation of mouse embryonic stem cells to insulin-producing cells Nat Protoc 2006 1 495 507 10.1038/nprot.2006.71 17406275

[B41] Chan KM Raikwar SP Zavazava N Strategies for differentiating embryonic stem cells (ESC) into insulin-producing cells and development of non-invasive imaging techniques using bioluminescence Immunol Res 2007 39 261 270 10.1007/s12026-007-0070-7 17917070

[B42] D’Amour KA Bang AG Eliazer S Kelly OG Agulnick AD Smart NG Moorman MA Kroon E Carpenter MK Baetge EE Production of pancreatic hormone-expressing endocrine cells from human embryonic stem cells Nat Biotechnol 2006 24 1392 1401 10.1038/nbt1259 17053790

[B43] Ku HT Chai J Kim YJ White P Purohit-Ghelani S Kaestner KH Bromberg JS Insulin-expressing colonies developed from murine embryonic stem cell-derived progenitors Diabetes 2007 56 921 929 10.2337/db06-0468 17395739

[B44] Miyazaki S Yamato E Miyazaki J Regulated expression of pdx-1 promotes in vitro differentiation of insulin-producing cells from embryonic stem cells Diabetes 2004 53 1030 1037 10.2337/diabetes.53.4.1030 15047618

[B45] Segev H Fishman B Ziskind A Shulman M Itskovitz-Eldor J Differentiation of human embryonic stem cells into insulin-producing clusters Stem Cells 2004 22 265 274 10.1634/stemcells.22-3-265 15153604

[B46] Shi Y Hou L Tang F Jiang W Wang P Ding M Deng H Inducing embryonic stem cells to differentiate into pancreatic beta cells by a novel three-step approach with activin A and all-trans retinoic acid Stem Cells 2005 23 656 662 10.1634/stemcells.2004-0241 15849173

[B47] Kahan B Magliocca J Merriam F Treff N Budde M Nelson J Browning V Ziehr B Odorico J Elimination of tumorigenic stem cells from differentiated progeny and selection of definitive endoderm reveals a Pdx1+ foregut endoderm stem cell lineage Stem Cell Res 2011 6 143 157 10.1016/j.scr.2010.10.003 21130058 PMC3040268

[B48] Lim SM Li X Schiesser J Holland AM Elefanty AG Stanley EG Micallef SJ Temporal restriction of pancreatic branching competence during embryogenesis is mirrored in differentiating embryonic stem cells Stem Cells Dev 2012 21 1662 1674 10.1089/scd.2011.0513 22034992

[B49] Brolen GK Heins N Edsbagge J Semb H Signals from the embryonic mouse pancreas induce differentiation of human embryonic stem cells into insulin-producing beta-cell-like cells Diabetes 2005 54 2867 2874 10.2337/diabetes.54.10.2867 16186387

[B50] Hansson M Tonning A Frandsen U Petri A Rajagopal J Englund MC Heller RS Hakansson J Fleckner J Skold HN Melton D Semb H Serup P Artifactual insulin release from differentiated embryonic stem cells Diabetes 2004 53 2603 2609 10.2337/diabetes.53.10.2603 15448090

[B51] Rajagopal J Anderson WJ Kume S Martinez OI Melton DA Insulin staining of ES cell progeny from insulin uptake Science 2003 299 363 12532008 10.1126/science.1077838

[B52] Kajiyama H Hamazaki TS Tokuhara M Masui S Okabayashi K Ohnuma K Yabe S Yasuda K Ishiura S Okochi H Asashima M Pdx1-transfected adipose tissue-derived stem cells differentiate into insulin-producing cells in vivo and reduce hyperglycemia in diabetic mice Int J Dev Biol 2010 54 699 705 10.1387/ijdb.092953hk 19757377

[B53] Karnieli O Izhar-Prato Y Bulvik S Efrat S Generation of insulin-producing cells from human bone marrow mesenchymal stem cells by genetic manipulation Stem Cells 2007 25 2837 2844 10.1634/stemcells.2007-0164 17615265

[B54] Li Y Zhang R Qiao H Zhang H Wang Y Yuan H Liu Q Liu D Chen L Pei X Generation of insulin-producing cells from PDX-1 gene-modified human mesenchymal stem cells J Cell Physiol 2007 211 36 44 10.1002/jcp.20897 17226789

[B55] Lin G Wang G Liu G Yang LJ Chang LJ Lue TF Lin CS Treatment of type 1 diabetes with adipose tissue-derived stem cells expressing pancreatic duodenal homeobox 1 Stem Cells Dev 2009 18 1399 1406 10.1089/scd.2009.0010 19245309 PMC2862049

[B56] Xu H Tsang KS Chan JC Yuan P Fan R Kaneto H Xu G The combined expression of Pdx1 and MafA with either Ngn3 or NeuroD improve the differentiation efficiency of mouse embryonic stem cells into insulin-producing cells Cell Transplant 2012 Epub ahead of print 10.3727/096368912X653057 22776709

[B57] Kubo A Stull R Takeuchi M Bonham K Gouon-Evans V Sho M Iwano M Saito Y Keller G Snodgrass R Pdx1 and Ngn3 overexpression enhances pancreatic differentiation of mouse ES cell-derived endoderm population PLoS One 2011 6 e24058 10.1371/journal.pone.0024058 21931641 PMC3172220

